# Primary Malignant Melanoma of the Urinary Bladder

**DOI:** 10.1155/2011/932973

**Published:** 2011-09-07

**Authors:** Jalal Eddine El Ammari, Youness Ahallal, Mohammed Jamal El Fassi, Moulay Hassan Farih

**Affiliations:** Department of Urology, University Hospital Center Hassan II-Fes, Morocco

## Abstract

*Introduction*. Primary melanoma of the urinary bladder is very rare. As far as we know, 19 cases have been reported worldwide, usually as case reports. *Case Presentation*. We present a 71-year-old male patient presented with a 2-month history of hematuria. Ultrasonography revealed a 5-cm-size mass located in the bladder trigone. A transurethral resection of the bladder tumor (TURBT) revealed a malignant melanoma. Evaluation for metastatic disease was negative. The patient deceased five months later before radical treatment could be performed. *Conclusion*. This is one more reported case of primary melanoma of the urinary bladder. The previously reported cases of bladder melanoma are reviewed. Therapy and prognosis are discussed.

## 1. Introduction

Malignant melanoma originating from the urinary bladder is very rare although urethral melanoma has been well described [1]. Melanoma of the bladder is most commonly secondary presentation of patients with widespread metastatic melanoma originating from the skin. Exclusion of other melanoma sites is mandatory to confirm primary bladder melanoma.

## 2. Case History

**Figure 1:**
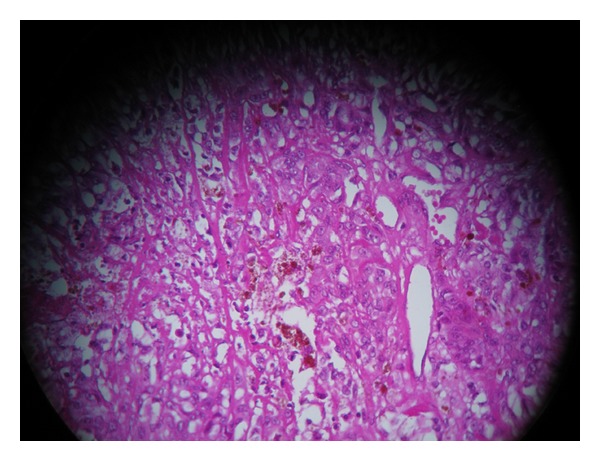
Section of tumor mass shows pigmented malignant melanocytes (HES ×20).

**Figure 2:**
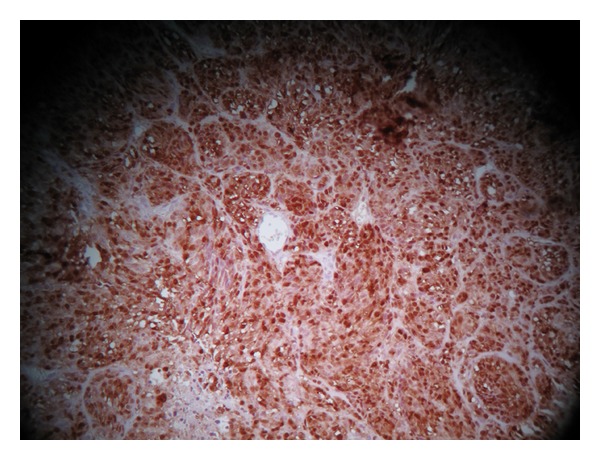
Tumor cells showing positive immunostaining to PS100 (HES ×20).

A 71-year-old Moroccan male presented with a 2 months history of gross hematuria. There was no past history of calculous disease or flank pain. He had been smoking 50 cigarettes a day for the past 40 years. There was no history of contact with aniline dyes or other chemicals. The physical and paraclinical examinations were normal. Ultrasonography revealed a 5-cm-size heterogeneous mass located in the bladder trigone. Cystoscopy showed a 50 × 40 × 10 mm darkly pigmented flat papillary lesion of the bladder trigone. A deep transurethral resection of the bladder has been, therefore, performed. The resection specimen examination showed pigmented malignant melanoma (Figures 1 and 2). Exhaustive investigation was undertaken to exclude other primary sites as the source of the melanoma. Careful dermatologic examination including a Wood's light examinations of the whole body and ophthalmologic evaluations were negative. Upper gastrointestinal and barium enema studies were negative. CT scan of the abdomen, including liver and spleen, was normal, as was CT scan of the brain and chest. Bone scan was negative. The diagnosis of primary malignant melanoma of the urinary bladder was made. The patient deceased five months later before any radical treatment could be performed. 

## 3. Discussion

Malignant melanoma of the genitourinary tract has been well documented. It is most commonly secondary to melanoma occurring in other sites. Numerous cases of primary melanoma of the urinary bladder have been previously reported. In such cases, a detailed patient history, careful examination of the patient's skin, and evaluation for other visceral primary sites are necessary to confirm the primary nature of the tumor [2].

The histogenesis of primary bladder melanoma is uncertain, and an origin from cells of the neural crest has been proposed [3].

In 1976, Ainsworth and associates were the first observers to carefully delineate criteria for defining primary malignant melanoma of the urinary bladder. These include (1) careful physical examination including the skin with Wood's light together with detailed history to exclude cutaneous melanoma, (2) exclusion of visceral melanoma following exhaustive evaluation, (3) pattern of recurrence consistent with primary melanoma of the urinary bladder, and (4) histologically proved primary atypical melanocytes [2].

Wheelock [4] was the first to report a primary melanoma of the bladder in 1942, then Su reported one case in 1962, followed by Anichkov and Nikonov [5] who reported 2 cases of primary bladder melanoma but in neither case were exhaustive studies undertaken to exclude other sites' primary melanoma [6–8]. The primary bladder melanoma is extremely rare, and probably none of those previously reported cases truly arose from the bladder.

Several treatments were proposed to deal with such a rare tumor. Transurethral resection, partial cystectomy, radical cystectomy, chemotherapy, and radiation therapy have been used to treat bladder melanoma [2, 6, 8, 9]. In all patients with localised tumor, radical surgery seems to be the therapy of choice, although to date none of the patients survived more than three years despite cystectomy characterizing the poor prognosis of the tumor [2, 6, 8, 9]. Since the tumor recur after local excision in many cases, adjuvant chemotherapy associated with radiotherapy may improve patients' survival [10]. Interferon is another alternative for the management of melanoma and showed 10% remission rate in metastatic cutaneous melanomas [11].

Despite this variety of treatments, the prognosis is bad. Ainsworth's patient was treated with radical cystectomy followed by chemotherapy and radiotherapy and developed local recurrence after 14 months [2]. In 2006, Pacella et al. reported an 82-year-old patient who presented a local recurrence and died nine months after the diagnosis. Lately, our patient died of widespread disease two months after diagnosis. However, Neiderberger reported 18 months disease-free survival in a primary bladder melanoma patient treated with radical cystectomy alone [8]. 

In all reported cases, the tumor was deeply invading the muscle which may explain the very bad prognosis [2, 6, 9].

## 4. Conclusion

Melanoma of the bladder is most commonly a secondary presentation of patients with widespread metastatic melanoma originating from the skin. We have shown that primary melanoma of the bladder is extremely rare, and all the criteria reported by Ainsworth should be met to confirm the diagnosis [2]. Treatment of the primary melanoma of the bladder should include radical surgery. The prognosis is guarded.
